# Dismal outcome of refractory or relapsing patients with myelodysplasia‐related acute myeloid leukemia partially alleviated by intensive chemotherapy

**DOI:** 10.1002/cam4.7003

**Published:** 2024-02-24

**Authors:** Harmony Leroy, Noémie Gadaud, Emilie Bérard, Emilie Klein, Isabelle Luquet, Jean‐Philippe Vial, Jean‐Baptiste Rieu, Nicolas Lechevalier, Suzanne Tavitian, Thibaut Leguay, Laetitia Largeaud, Audrey Bidet, Eric Delabesse, Audrey Sarry, Anne‐Charlotte de Grande, Christian Récher, Arnaud Pigneux, Sarah Bertoli, Pierre‐Yves Dumas

**Affiliations:** ^1^ CHU Bordeaux, Service d'Hématologie Clinique et de Thérapie Cellulaire Bordeaux France; ^2^ Service d'Hématologie Centre Hospitalier Universitaire de Toulouse Institut Universitaire du Cancer de Toulouse‐Oncopole Toulouse France; ^3^ Centre Hospitalier Universitaire de Toulouse, Service d'Epidémiologie, CERPOP, Inserm, Université Toulouse III Paul Sabatier Toulouse France; ^4^ CHU Bordeaux, Laboratoire d'Hématologie Biologique Bordeaux France; ^5^ Laboratoire d'Hématologie Centre Hospitalier Universitaire de Toulouse, Institut Universitaire du Cancer de Toulouse‐Oncopole Toulouse France; ^6^ Université de Bordeaux, Bordeaux, Institut National de la Santé et de la Recherche Médicale Bordeaux France

**Keywords:** acute myeloid leukemia, azacitidine, intensive chemotherapy, myelodysplasia‐related changes, primary induction failure, refractoriness, relapse

## Abstract

**Background:**

Acute myeloid leukemia (AML) with myelodysplasia‐related characteristics is a heterogeneous subset of AML that has been challenged throughout the history of myeloid malignancies classifications, considered to have similar outcomes as intermediate‐ or adverse‐risk AML depending on the subgroup. However, little is known about the fate of these patients in refractory or relapsed situation (R/R) after first line therapy.

**Methods:**

A large series of R/R AML patients, recorded in the French DATAML registry, have received either intensive chemotherapy (ICT), azacitidine (AZA) as single agent, or best supportive care (BSC). A cohort of 183 patients (median age 63‐year‐old) with what was called at the time AML‐MRC has been explored, and data are reported here.

**Results:**

Patient status was refractory for 93, while 90 had relapsed. Respectively, 88, 34, and 61 were included in the three treatment arms. The median OS of the whole cohort was 4.2 months (95%CI: 3.1–5.6) with a mean 1‐year overall survival of 24% ± 3.2%. There was no significant survival difference between refractory and relapsed patients. The BSC group had overall a significantly worse outcome (*p* = 0.0001), and this remained true in both refractory (*p* = 0.01) and relapsed (*p* = 0.002) patients. Similar survivals were observed in both groups comparing ICT and AZA.

**Conclusions:**

These data, reporting about an ill‐explored population, indicate the poor prognosis of this condition where both ICT and AZA can be proposed. The latter, which was demonstrated here to be a feasible option, should be added to new targeted therapies.

## 1 INTRODUCTION

In 2001, acute myeloid leukemia (AML) with multilineage dysplasia was formally integrated in the World Health Organization (WHO) classification of tumors of hematopoietic and lymphoid tissues as a specific entity.^1^ It was defined for de novo patients as the presence of bone marrow (BM) dysplasia in at least 50% of the cells in two or more myeloid lineages. It was also considered for patients with a previous history of myelodysplastic syndrome (MDS) or MDS/myeloproliferative neoplasia (MDS/MPN). Later, the WHO 2008 classification^2^, published in 2009, introduced the notion of AML with myelodysplasia‐related changes (AML‐MRC), and some specific MDS‐related cytogenetic abnormalities were considered as additional criteria. The AML‐MRC entity was redefined by the WHO 2016^3^ classification on the basis of molecular markers, and the presence of multilineage dysplasia alone thus did no longer classify a case as AML‐MRC if a good prognosis *NPM1* or biallelic *CEBPA* mutation was present.^3^ Finally, in the most recent 5th WHO classification, the term AML‐MR was coined.^4^ In this heterogeneous disease, patients with AML and multilineage dysplasia alone (AML‐MLD‐sole) have been shown to display a better outcome than those with cytogenetic abnormalities (AML‐MRC‐C)^5^ or having a previous history of MDS/MPN (AML‐MRC‐S), treated or not.^6^

In 2009, the pivotal study AZA‐001 demonstrated the efficacy of azacitidine (AZA), compared to conventional care regimens (CCR), in the treatment of higher‐risk MDS, refractory anemia with excess blasts, refractory anemia with excess blasts in transformation or chronic myelomonocytic leukemia with low bone marrow blast counts.^7^, ^8^ In 2015, the AZA‐AML‐001 study confirmed that AZA increased the median overall survival (OS) by 3.8 months versus CCR in newly diagnosed AML with bone marrow blast counts over 30% in patients older than 65 years of age.^9^ Interestingly, a subgroup analysis of this trial, focusing on AML‐MRC patients, showed that the median OS was significantly prolonged with AZA versus CCR (hazard ratio 0.74 [95%CI 0.57, 0.97]). This was particularly true for patients with intermediate‐risk cytogenetics who reached a median OS of 16.4 months with AZA versus 8.9 months with CCR.^10^ AZA thus became the preferred treatment for patients with AML‐MRC not eligible for intensive chemotherapy (ICT), particularly older patients with intermediate‐risk cytogenetics AML‐MRC.^10^ Meanwhile, in a randomized phase II study of patients aged from 60 to 75 years old with secondary AML, CPX‐351, a liposomal combination of cytarabine and daunorubicin, demonstrated a better antileukemic activity than the classical 3 + 7 combination, making CPX‐351 a new option for these poor prognosis patients.^11^ This led to a randomized phase III study^12^ comparing CPX‐351 to 7 + 3, for the treatment of adults with newly diagnosed therapy‐related AML or AML‐MRC. A significant improvement of the remission rate was confirmed for patients who received CPX‐351 (47.7% vs 33.3%; *p* = 0.016) as well as a better median OS (9.5 months vs 5.9 months; *p* = 0.003). Since then, more recently developed drugs have been tested in the context of R/R AML,^13^, ^14^, ^15^, ^16^ yet without singling out patients with MRC‐AML.

An interesting point that has not been addressed in these studies would be to explore the outcome of the subgroup of AML‐MRC patients in a refractory or relapse (R/R) situation. The retrospective study presented here was thus performed to compare the efficacy of ICT or AZA compared to best supportive care (BSC) in such R/R AML‐MRC patients.

## 2 METHODS

### 2.1 Patients and treatments

This study retrospectively included patients with AML‐MRC according to the 2016 WHO classification,^3^ diagnosed between January 1, 2007, and December 31, 2016 and enlisted in the DATAML registry maintained in two French medical centers (Bordeaux and Toulouse University Hospitals). Written informed consent was obtained in accordance with the Declaration of Helsinki, allowing for the collection of clinical data in an anonymized database. After evaluation and validation by the data protection officer and according to the General Data Protection Regulation, it is covered by the MR‐004, CNIL number 2206723v0, so stating of the current study is exempt from national Ethics Committee. Cytogenetic risk was assessed according to the Medical Research Council classification.^17^ Patients over 18 years of age were included if they had been treated by ICT in front line and were in primary induction failure (including after a second induction if BM blasts were over 5% at day [d] 15), or in relapse after having achieved a complete remission (CR) or CR with incomplete hematological recovery (CRi). They also should have received salvage treatment by a second line of ICT, AZA or BSC.

Relapse was defined as BM blasts ≥5%, reappearance of blasts in the blood or development of extramedullary disease.^18^ The ICT salvage regimens used were based on (i) single‐agent cytarabine (high‐dose: 3 g/m^2^/12 h, d1‐4, or intermediate dose: 1–1.5 g/m^2^/12 h, d1‐4, or 1 g/m^2^/d, d1‐5), (ii) combination of an anthracycline plus cytarabine (daunorubicin 60 mg/m^2^/d, d1‐3, or idarubicin 12 mg/m^2^/d, d1‐3, or amsacrine 200 mg/m^2^/d, d1‐3 + cytarabine 1.5–3 g/m^2^/12 h, d1‐4), or (iii) the FLAG‐IDA regimen (fludarabine 30 mg/m^2^/d, d1‐5 + cytarabine 2 g/m^2^/d, d1‐5 + idarubicin 10 mg/m^2^/d, d1‐3 + G‐CSF 5 μg/kg/d, d1‐5). The choice between these different options was made on a case‐by‐case basis, depending on each patient's performance status, previous treatment history, disease characteristics and time to relapse. AZA was administered to 34 patients; as a single agent (75 mg/m^2^/d, d1‐7 or 5 + 2) in 28 patients, in combination with lenalidomide in 5 patients, or with all‐trans retinoic acid in one patient.^19^ No patient received decitabine, as this drug is not available in France. Response to treatment was defined according to the European LeukemiaNet 2017 criteria^18^ for ICT and to IWG 2006 criteria^20^ for AZA. Neither of the two centers use antibioprophylaxis for patients receiving intensive chemotherapy, AZA as single agent or BSC. Patients receiving intensive chemotherapy receive antifungal prophylaxis during neutropenic period, patients receiving AZA single agent or BSC do not receive antifungal prophylaxis.

### 2.2 Statistical analyses

Statistical analyses were performed using STATA statistical software, release 14.2 (STATA Corp., College Station, TX) and Medcalc (Ostende, Belgium). All reported *p* values were two‐sided and a threshold of 0.05 was considered for statistical significance. Patient characteristics were reported using numbers and frequencies for qualitative data. For quantitative data, medians, inter‐quartile ranges (IQR) and ranges (minimum maximum) were used. Categorical variables were compared between groups using the Chi^2^ test (or Fisher's exact test when necessary). Comparison of OS and PFS was assessed using log‐rank test and Kaplan–Meier graphical representation in univariate analyses.

## 3 RESULTS

### 3.1 Study population at baseline

Examination of the DATAML registry for the period considered identified a total of 662 R/R AML patients.^21^ Of them, 183 AML‐MRC patients (27.6%) fulfilled inclusion criteria for this study. Their characteristics are presented in Table 1. The median age of the cohort was 63‐year‐old (range, 18–77), including 132 patients (72.1%) over age 60. Extramedullary involvement and leukostasis were observed in 46 (25.1%) and 4 patients, respectively. The median white blood cell (WBC) count was 4.1 × 10^9^/L (IQR, 2.0–12.8) at diagnosis. There were 103 (56.3%) cases of de novo AML‐MRC. Patients could be subdivided in 123 (67.2%) AML‐MRC‐C, 32 (17.4%) AML‐MRC‐S and 28 (15.3%) AML‐MLD‐sole. None of the patients displayed *NPM1* or biallelic *CEBPA* mutations. As shown in Figure S1, most patients had only one criterion allowing for AML‐MRC classification and a single patient displayed all 3 characteristics. Table S1 also reports the specific cytogenetic features of the cohort. Induction therapy was daunorubicin‐based for 47 (25.7%) patients and idarubicin‐based for 129 (70.5%) as detailed in Table S2. The remaining 7 patients (3.8%) received ICT with an experimental drug in a clinical trial.

**TABLE 1:** Baseline patient characteristics at diagnosis.

	Total *n* = 183
Age at diagnosis (years)	
Median (IQR)	63.0 (56.0–67.0)
ECOG at diagnosis: *n* (%)	
0–1	144 (82.8)
≥2	30 (17.2)
WBC at diagnosis (×10^9^/L)	
Median (IQR)	4.1 (2.0–12.8)
AML status: *n* (%)	
De novo	103 (56.3)
Secondary AML	80 (43.7)
Cytogenetic risk: *n* (%)	
Favorable	0 (0.0)
Intermediate	60 (32.8)
Adverse	123 (67.2)
ELN 2010 prognosis: *n* (%)	
Favorable	3 (1.7)
Intermediate‐I/II	48 (27.8)
Adverse	122 (70.5)
FLT3‐ITD: *n* (%)	
Mutation	9 (9.7)
No mutation	84 (90.3)
NPM1: *n* (%)	
Mutation	6 (7.1)
No mutation	78 (92.9)
CEBPA: *n* (%)	
Mutation	3 (5.7)
No mutation	50 (94.3)

Abbreviations: IQR, interquartile range; WBC, white blood cells; ECOG, Eastern Cooperative Oncology Group; AML, acute myeloid leukemia; ELN, European LeukemiaNet.

Response to chemotherapy after induction was primary induction failure in 93 (50.8%) patients while 90 (49.2%) reached CR/CRi then relapsed. For the latter, the median time to relapse was 9.7 months (IQR, 2.4–66.8).

### 3.2 Study population at R/R

Patient characteristics at R/R are reported in Table 2. Salvage ICT was prescribed to 88 patients (48.1%) while 34 (18.6%) received AZA and 61 (33.3%) had BSC only (Figure S2). The median age of the whole cohort at R/R was 63.0 years (range, 18–77) and increased according to salvage therapy groups (60‐, 62‐, and 67‐year‐old respectively). A performance status of 0–1 was observed for 124 patients (67.7%), also displaying different frequencies according to salvage therapy (77.2%, 73.5%, and 50.8%, respectively). There was no significant difference between the three regimens regarding the distribution of AML‐MRC‐C (67.0%, 64.7%, and 68.8%), AML‐MRC‐S (20.5%, 20.5%, and 34.4%) or AML‐MLD‐sole (21.5%, 26.4%, and 31.1%). Significantly more patients receiving ICT were refractory (65.9%) than in relapse (34.1%, *p* < 0.0001). These proportions were 26.5%/76.5% for AZA and 44.3%/55.7% for BSC (*p* = 0.0025 and 0.08). Of note, patients in the BSC arm were older and had more comorbidities.

**TABLE 2:** R/R patient characteristics at inclusion according to treatment arms.

	ICT *n* = 88 (48.1%)	AZA *n* = 34 (18.6%)	BSC *n* = 61 (33.3%)	Total *n* = 183 (100%)
Age (years)				
Median (IQR)	60.0 (49.0–64.0)	62.0 (56.5–66.0)	67.0 (62.0–72.0)	63.0 (56.0–67.0)
Range	18.0–73.0	42.0–77.0	34.0–76.0	18.0–77.0
ECOG: *n* (%)				
0–1	68 (77.2)	25 (73.5)	31 (50.8)	124 (67.7)
≥2	16 (18.1)	5 (14.7)	26 (42.6)	47 (25.6)
AML‐MRC status: *n* (%)				
AML‐MRC‐C	59 (67.0)	22 (64.7)	42 (68.8)	123 (67.2)
AML‐MRC‐S	18 (20.5)	7 (20.5)	21 (34.4)	46 (25.1)
AML‐MLD‐sole	19 (21.5)	9 (26.4)	8 (13.1)	36 (19.6)
Refractory or relapse: *n* (%)				
Primary induction failure	58 (65.9)	8 (26.5)	27 (44.3)	93 (50.9)
Relapse	30 (34.1)	26 (76.5)	34 (55.7)	90 (49.1)

Abbreviations: ICT, intensive chemotherapy; AZA, azacitidine; BSC, best supportive care. IQR, interquartile range; ECOG, Eastern Cooperative Oncology Group; AML, acute myeloid leukemia; MRC, myelodysplasia‐related changes; AML‐MRC‐C, cytogenetic abnormalities; AML‐MRC‐S, previous history of MDS/MPN; AML‐MLD‐sole, multilineage dysplasia alone.

### 3.3 Clonal evolution

**FIGURE 1:**
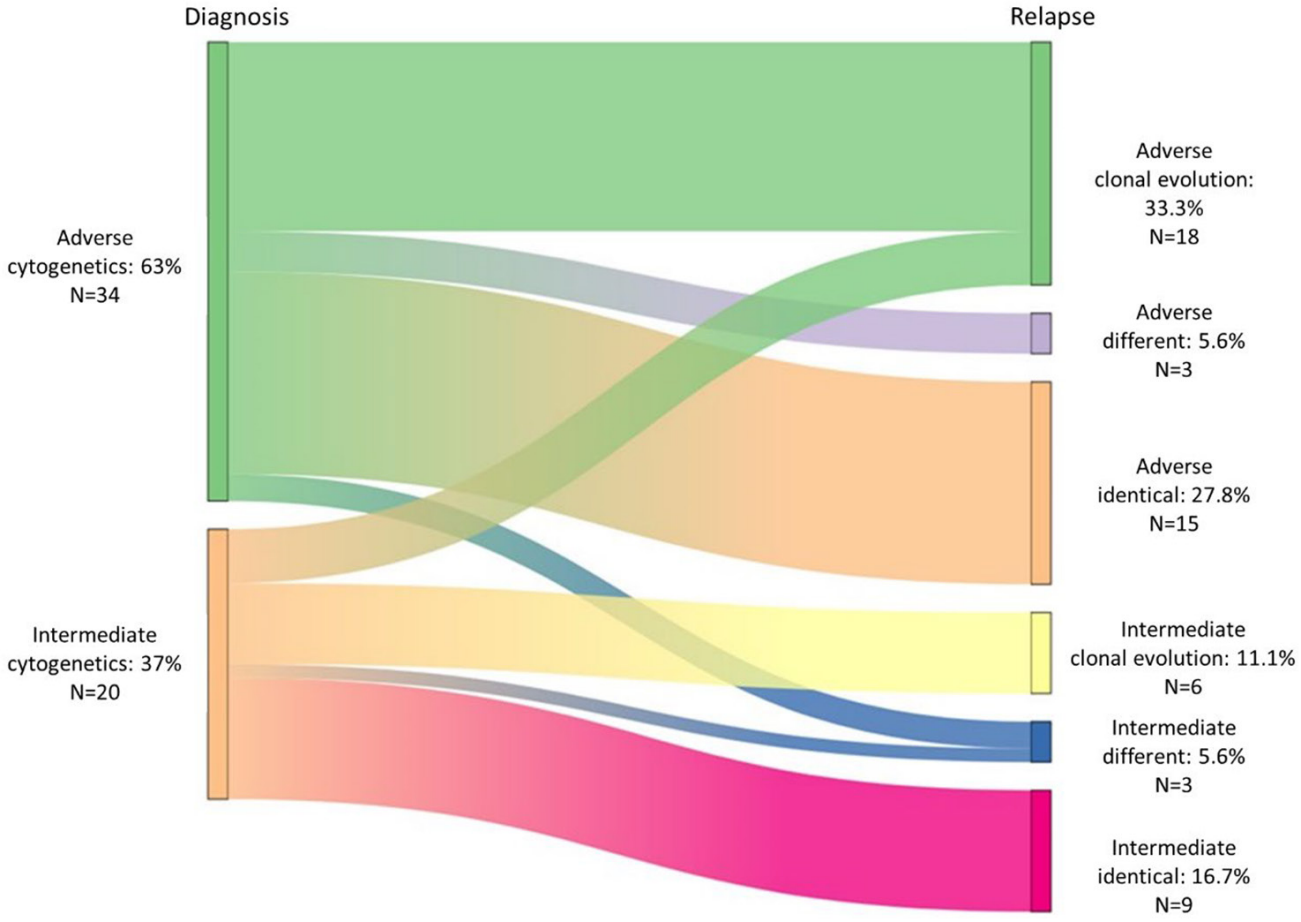
Clonal evolution upon treatment. For the 54 patients who benefited from a karyotype at inclusion and after treatment, data comparison allowed to identify three patterns of respectively (i) acquisition of a new abnormality in a previously known clone (clonal evolution), (ii) appearance of new clones (different), or (iii) no change (identical). Of note, this led a few patients to change of cytogenetic risk, respectively from adverse (green) to intermediate and vice versa (Orange).

Among the cohort of 90 relapsing patients, 54 benefited from paired karyotypes at diagnosis and relapse. This showed that 24 (44.4%) were identical while 30 (55.6%) were different with or without clonal evolution (Figure 1). More precisely, among 34 karyotypes with adverse cytogenetics, 15 (44.1%) remained identical, and of 20 karyotypes with intermediate cytogenetics at relapse, 9 (45%) were identical.

### 3.4 Response to salvage treatment and outcomes

**FIGURE 2:**
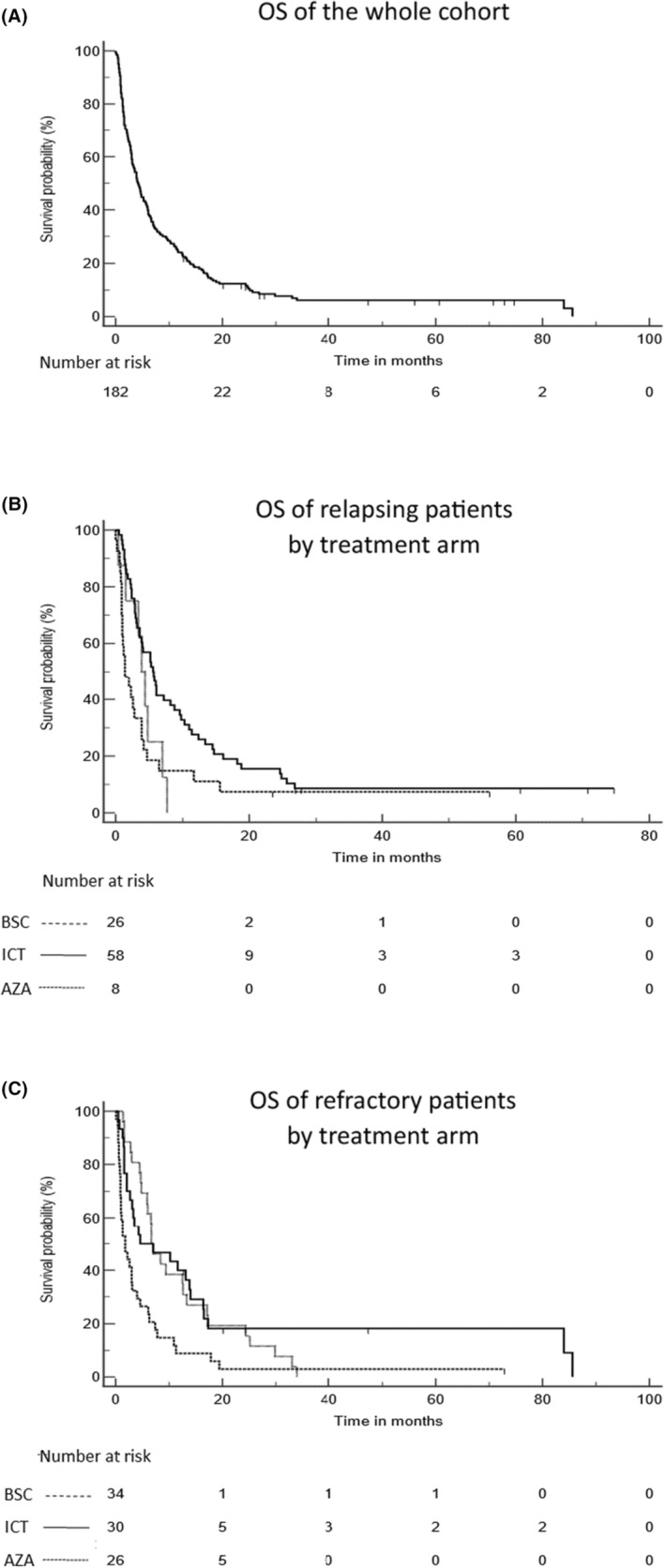
Overall survival. The data presented are of the whole population (A) and comparing the three treatment arms in relapsed (B), or refractory (C) patients. ICT (solid line); AZA (dotted line) and BSC (dashed line).

For the whole cohort, CR/CRi was obtained after salvage treatment for 18 patients (9.8%). They were 16 (18.2%) CR/CRi in the ICT group and 2 (5.9%) in the AZA group (median 3 cycles, range 1–10). No response was obtained after BSC. Allo‐HSCTs have been performed for more patients (*n* = 32; 36.4%) after ICT than after AZA (*n* = 3; 8.8%). OS according to the whole group and the type of treatment in refractory and relapsed patients are shown in Figure 2 and Table 3. With a median follow up of 4.2 months (range 0.03–72.9) for the whole cohort (37.6 months for alive patients, range 12.7.‐74.7), median OS was 4.2 months (95%CI: 3.1–5.6) with a 12‐month OS at 24% ± 3.2%. This median OS was similar, whether patients were refractory (3.9 months, 95%CI 2.9–5.3) or had relapsed (4.6 months, 95%CI 3.0–6.7, *p* = 0.6, Figure S3A). There was however a significant difference in OS depending on the type of treatment received, considering the whole cohort (*p* = 0.0001, Figure S3B). The difference was related to the poor outcome of BSC patients (median OS 1.4 months, 95%CI 0.9–3.9 in refractory patients and 1.8 months, 95%CI 0.9–3.1 in relapsed patients). Conversely, there was no difference according to ICT or AZA regimen, neither in refractory (*p* = 0.06) nor relapsed (*p* = 0.65) patients (Table 4). There was no difference either when comparing patients who qualified for MRC by only one criterion (Figure S4), yet patients with MRC‐sole had a significantly better outcome (*p* = 0.03) than patients with MRC‐S. There was no difference either when comparing PFS by treatment arm (Figure S5).

**TABLE 3:** Overall survival by subgroups.

Population	Median OS (95%CI) months	Mean ± SD 12 months OS %	*p* Value
Overall	4.2 (3.1–5.6)	2.4 ± 0.3	
RR status			
Refractory	3.9 (2.9–5.3)	20.4 ± 4.2	0.6
Relapsed	4.6 (3.0–6.7)	27.8 ± 4.7	
Treatment			
ICT	5.6 (3.6–8.4)	31.8 ± 4.9	0.01
AZA	6.6 (4.5–8.4)	29.4 ± 7.8	
BSC	1.8 (1.0–2.8)	9.8 ± 3.8	
Treatment refractory			
ICT	5.6 (3.6–8.8)	11.1 ± 6.0	0.01
AZA	3.9 (0.4–7.0)	0	
BSC	1.4 (0.9–3.9)	0	
Treatment relapsed			
ICT	4.6 (2.7–13.8)	40.0 ± 8.9	0.002
AZA	6.7 (4.8–12.6)	38.5 ± 9.5	
BSC	1.8 (0.9–3.1)	8.8 ± 4.9	

Abbreviations: ICT, intensive chemotherapy; AZA, azacitidine; BSC, best supportive care; OS, overall survival; SD, standard deviation; R/R, relapsed or refractory.

**TABLE 4:** Overall survival significance comparing treatment arms two by two (*p* values).

Compared treatments	Refractory patients	Relapsed patients
ICT vs. AZA	0.06	0.65
ICT vs. BSC	0.005	0.005
AZA vs. BSC	0.58	0.0045

Abbreviations: ICT, intensive chemotherapy; AZA, azacitidine; BSC, best supportive care.

Of note, 93% of the patients ultimately died of AML or related complications (infections or graft vs host disease). One committed suicide, two succumbed to strokes and one from a secondary cancer (lymphoma).

## 4 DISCUSSION

While the subgroup of patients with AML‐MRC has globally a poorer prognosis than non‐MRC AML patients in first line therapy, little is known of the outcome of these poor responders at later stages of the disease. The aim of this original study was to specifically examine the outcomes of R/R AML‐MRC patients. It confirms that the prognosis remains dismal in such cases, whether patients were refractory or had relapsed. Compared to BSC, the use of ICT however resulted in a significantly better prognosis in both groups, while AZA provided a better survival to relapsed patients.

Because the study was performed in a time frame when molecular analyses were not as exhaustive as nowadays, it was not possible to apply the most recent WHO classification^4^ and exactly determine how many of these patients would actually have fulfilled the criteria of the new denomination “AML, myelodysplasia‐related (AML‐MR).” It is however unlikely that this would have dramatically changed the results of this work.

The major comparisons available between this cohort of R/R AML‐MRC patients and published studies relate to baseline patient characteristics and responses to first line therapy. Here, more than 88% of the patients were classified as MRC‐AML based on only one criterion with respective proportions of 67.2% of AML‐MRC‐C, 17.4% patients with a history of MDS (AML‐MRC‐S) and 15.3% of AML‐MLD‐sole. Montalban‐Bravo et al.^6^ reported almost similar proportions of 59% of patients classified on the basis of cytogenetics, 23% of AML‐MLD‐sole and 18% with a history of MDS or MDS/MPN. This differs from the cohort of Xu et al.^18^ who reported 49% of AML‐MLD‐sole, 20% of AML‐MRC with a previous history of MDS/MPN and only 18.3% of AML‐MRC‐C, while the remaining 12.7% of patients had various histories.

Most of the patients in this cohort had a complex karyotype or monosomies, making them of very poor prognosis. These data are consistent with the cohorts reported by Montalban‐Bravo et al.^6^ and Kaivers et al.^22^ Moreover, between diagnosis and relapse, broadly 50% of the patients presented with a clonal evolution or different cytogenetics. A similar pattern was reported by Kern et al.,^23^ who found 60% of additional abnormalities at relapse in AML patients with an unfavorable karyotype at diagnosis. In the latter study, clonal evolution resulted in a significantly worse response to treatment at relapse and worse outcomes compared to patients with a stable karyotype.

The very low proportion of patients with more than one MRC factor, both in the first line series of Montalban‐Bravo et al.^6^ and Kaivers et al.^22^ and in the present cohort of R/R patients is quite striking. It illustrates the fact that AML‐MRC is a heterogeneous group of diseases with unfavorable prognosis yet different pathophysiology. The importance of molecular abnormalities to delineate this subset of AML was indeed suggested by Lindsley et al.^24^ It has recently been integrated in the WHO 2022 classification,^21^ as mentioned above, as well as in the International Consensus Classification (MDS/AML with myelodysplasia‐related gene mutation)^25^ and in the most recent ELN risk classification.^26^

Response to first line therapy was obtained 49.2% of the patients. Most of them had received ICT and these results are thus similar to those reported by Montalban‐Bravo et al.^6^ in their cohort of 415 newly diagnosed AML‐MRC patients where 29% (120) received ICT, of whom 51% reached CR. In the study by Xu et al.,^27^ CR was achieved for 37.4% of the patients with only one induction and increased up to 60.9% after salvage. In the cohort reported here, the median delay before relapse after front line therapy was 8 months, slightly better than the respective 6.9 months and 5 months of these authors.^5^, ^27^

Salvage therapy allowed to reach CR2/CRi2 in only 9.8% of these R/R AML‐MRC patients, essentially with ICT, while AZA performed more poorly. Of note, most survivors were able to receive allo‐HSCT, nearly all of them after ICT, suggesting a potentially effective bridge to transplant with this strategy. These data can be compared to those reported by Stahl et al.^28^ in a series of 655 R/R AML patients. Although the subgroup of AML‐MRC subjects was not identified in this study, AZA allowed to reach CR/CRi for 16.3% of the patients after a median of 3 AZA cycles and yielded an OS of 6.8 months (range 6–8.5).

This study has limitations, essentially because of its retrospective nature. Indeed, molecular data are lacking, that would have helped to better classify the patient. For the same reason, newly developed and potentially more efficient drugs are now available, that begin to be tested in this context.^13^, ^14^, ^15^, ^16^, ^17^

In conclusion, the BSC group here confirms the dismal prognosis of AML‐MRC patients. Although ICT and allo‐HSCT appear currently to be the best choice for R/R AML‐MRC patients, AZA could be considered a feasible option for patients ineligible to ICT. It might represent a companion of choice in future combination schedules, in line with recent publications in first line therapy.^29^, ^30^ This would be an attractive option for this population of patients with an obvious unmet medical need.

## AUTHOR CONTRIBUTIONS

**Harmony Leroy:** Data curation (equal); investigation (lead). **Noémie Gadaud:** Data curation (equal); investigation (equal). **Emilie Bérard:** Methodology (lead). **Emilie Klein:** Resources (equal). **Isabelle Luquet:** Resources (equal). **Jean‐Philippe Vial:** Resources (equal). **Jean‐Baptiste Rieu:** Resources (equal). **Nicolas Lechevalier:** Resources (equal). **Suzanne Tavitian:** Resources (equal). **Thibaut Leguay:** Resources (equal). **Laetitia Largeaud:** Resources (equal). **Audrey Bidet:** Resources (equal). **Eric Delabesse:** Resources (equal). **Audrey Sarry:** Investigation (equal). **Anne‐Charlotte de Grande:** Investigation (equal). **Christian Récher:** Supervision (equal). **Arnaud Pigneux:** Supervision (equal). **Sarah Bertoli:** Conceptualization (lead); data curation (lead); formal analysis (lead); methodology (equal); project administration (equal); supervision (equal). **Pierre‐Yves Dumas:** Conceptualization (lead); data curation (equal); formal analysis (equal); investigation (equal); methodology (lead); project administration (lead); supervision (lead); writing – original draft (lead).

## CONFLICT OF INTEREST STATEMENT

Harmony Leroy, no conflict of interest; Noémie Gadaud, no conflict of interest; Emilie Bérard, no conflict of interest; Emilie Klein, no conflict of interest; Isabelle Luquet, no conflict of interest; Jean‐Philippe Vial, no conflict of interest; Jean‐Baptiste Rieu, no conflict of interest; Nicolas Lechevalier, no conflict of interest; Tavitian, no conflict of interest; Thibaut Leguay, no conflict of interest; Laetitia Largeaud, no conflict of interest; Audrey Bidet, no conflict of interest; Eric Delabesse, no conflict of interest; Audrey Sarry, no conflict of interest; Anne‐Charlotte de Grande, no conflict of interest; Christian Récher: report grants, personal fees and non‐financial support from Abbvie; grants from Amgen; grants, personal fees and non‐financial support from Astellas; grants, personal fees and non‐financial support from BMS; grants, personal fees and non‐financial support from Jazz Pharmaceuticals; grants from Iqvia; personal fees and non‐financial support from Novartis; grants from MaatPharma; personal fees and non‐financial support from Servier; and personal fees from Takeda. Arnaud Pigneux: Grant/Research Support: Astellas and Roches; Speaker's Bureau: AbbVie, Astellas, Gilead, Pfizer, Roche and Sanofi; Consultant: AbbVie, Agios, BMS, Gilead, Jazz Pharma, Novartis, Pfizer, Roche and Takeda. Sarah Bertoli: Jazz Pharmaceuticals, Daiichi‐Sankyo, Sanofi, Astellas, BMS, Abbvie and Pfizer. Pierre‐Yves Dumas: Daiichi‐Sankyo, Jazz Pharmaceutical, Astellas, Abbvie, Celgene and Janssen.

## Supporting information


Appendix S1.

## Data Availability

Some de‐identified data will be shared with other researchers upon reasonable request to the corresponding authors (pierre-yves.dumas@u-bordeaux.fr). The sharing will require a detailed proposal to the study investigators, and a data transfer agreement must be signed.
