# Supplementary material for: Dismal outcome of refractory or relapsing patients with myelodysplasia‐related acute myeloid leukemia partially alleviated by intensive chemotherapy
Source: Cancer Med. 2024 Feb 24;13(3):e7003. doi: 10.1002/cam4.7003 (PMC10891460; doi:10.1002/cam4.7003)

**Supplemental data**

**Dismal outcome of refractory or relapsing patients with myelodysplasia-related acute myeloid leukemia partially alleviated by intensive chemotherapy**

Harmony Leroy^1^* M.D., Noémie Gadaud^2^* M.D., Emilie Bérard^3^ M.D., Emilie Klein^5^ Pharm.D., Isabelle Luquet^4^ Pharm.D., Jean-Philippe Vial^5^ Pharm.D., Jean-Baptiste Rieu^4^ Pharm.D., Nicolas Lechevalier^5^ Pharm.D., Suzanne Tavitian^2^ M.D., Thibaut Leguay^1^ M.D., Laetitia Largeaud^4^ Pharm.D.-Ph.D., Audrey Bidet^5^ Pharm.D., Eric Delabesse^4^ Pharm.D.-Ph.D., Audrey Sarry^2^, Anne-Charlotte de Grande^1^, Christian Récher^2^ M.D.-Ph.D., Arnaud Pigneux^1,6^ M.D.-Ph.D., Sarah Bertoli^2^# M.D.-Ph.D., Pierre-Yves Dumas^1,6^# M.D.-Ph.D.

**Supplemental Table S1**. Cytogenetic characteristics.

|  | **AML-MRC-C**  **N=123 ^a,c^** | **AML-MRC-S**  **N=32 ^b,c^** | **AML-MLD-sole**  **N=28** |
| --- | --- | --- | --- |
| **Complex karyotype (≥3 abnormalities)** | 90 (73.1) | 6 (18.7) | 0 (0.0) |
| **Unbalanced abnormalities** | 92 (74.8) | 10 (31.2) | 9 (32.0) |
| -7/del(7q) | 51 (41.5) | 3 (9.3) | 0 (0.0) |
| del(5q)/t(5q) | 55 (44.7) | 6 (18.7) | 0 (0.0) |
| i(17q)/t(17p) | 7 (5.6) | 1 (3.1) | 0 (0.0) |
| -13/del(13q) | 8 (6.5) | 0 (0.0) | 0 (0.0) |
| del(11q) | 6 (4.8) | 2 (6.2) | 0 (0.0) |
| del(12p)/t(12p) | 2 (1.6) | 1 (3.1) | 0 (0.0) |
| idic(X)(q13) | 2 (1.6) | 0 (0.0) | 0 (0.0) |
| **Balanced abnormalities** | 3 (2.4) | 0 (0.0) | 2 (7.3) |
| **Normal karyotype** | 0 (0.0) | 18 | 17 (60.7) |

AML-MRC-C, acute myeloid leukemia classified as myelodysplasia related changes by cytogenetics; AML-MRC-S, AML-MRC by previous history of myelodysplastic syndrome; AML-MLD-sole, AML-MRC by multilineage dysplasia in the absence of *NPM1* or biallelic mutation of *CEBPA*

^a^ 107 patients classified by cytogenetics, 13 by cytogenetics and previous history of MDS, 2 by cytogenetics and multilineage dysplasia, 1 by all 3 characteristics.

^b^ 27 patients classified by previous history of MDS, 5 by previous history of MDS and multilineage dysplasia

^c^ Some patients had complex karyotype with unbalanced abnormalities

**Supplemental Table S2**. Front line induction chemotherapy according to the R/R treatment arms.

|  | **ICT** | **AZA** | **BSC** |
| --- | --- | --- | --- |
| Idarubicin-based  Idarubicin-based + CCNU | 21  37 | 6  17 | 17  31 |
| Daunorubicin-based  Others | 28  2^a^ | 11  0 | 11  2^b^ |

ICT, intensive chemotherapy; AZA, azacitidine; BSC, best supportive care; DA : daunorubicin; CCNU, lomustine; CYTA, cytarabine, LEN : lenalidomide; GO : gemtuzumab-ozogamycin.

^a^ CYTA+CCNU, CYTA+Amonafide

^b^ CYTA

**Supplemental Figure legends**

Figure S1

Venn diagram of criteria qualifying patients for AML with myelodysplasia-related characteristics.

Figure S2

Flow chart.

Figure S3

Overall survival of refractory vs. relapsed MRC patients (A) and according to the type of treatment for the whole cohort (B).

Figure S4

Comparison of overall survival according to the type of MRC, for patients with only one criterion, *i.e.* MLD-sole, adverse karyotype (MRC-C) or antecedents of MDS/MPN (MRC-S). Comparison of the three groups is not statistically significant, but the comparison of MLD-sole vs. MRC-S shows a significantly worse outcome for the latter. One year OS is respectively 39.3 + 9.2%, 23.4 + 4.0% and 14.8 + 6.8%.

Figure S5

Progression free survival of R/R MRC patients according to the type of treatment for the whole cohort.


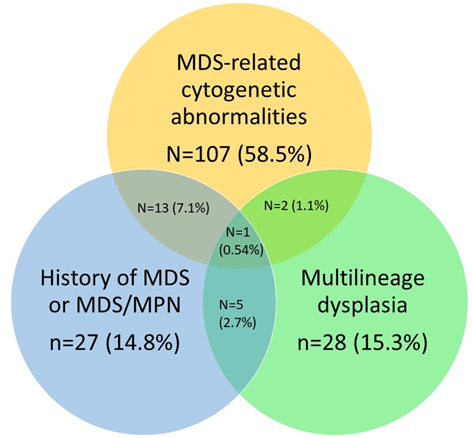
Figure S1.

Figure S2.


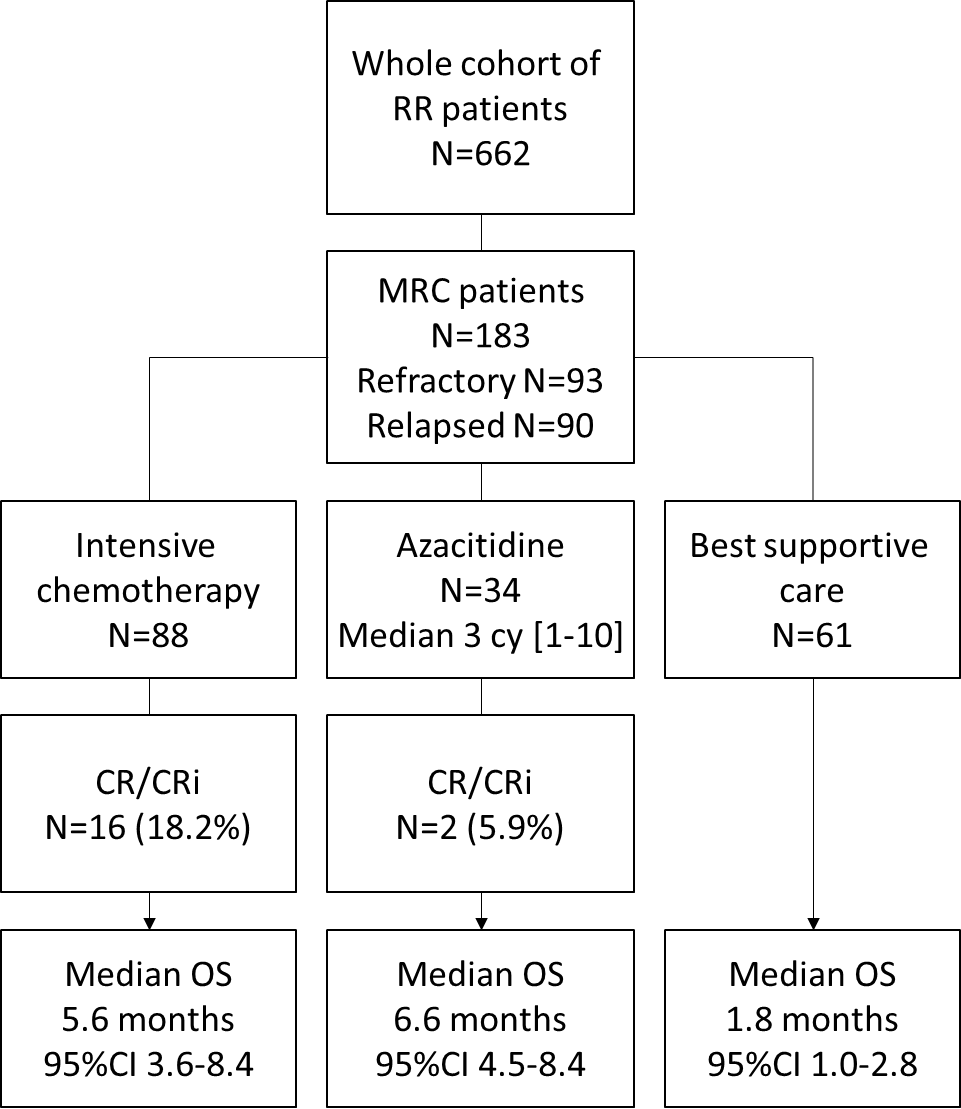


Figure S3.


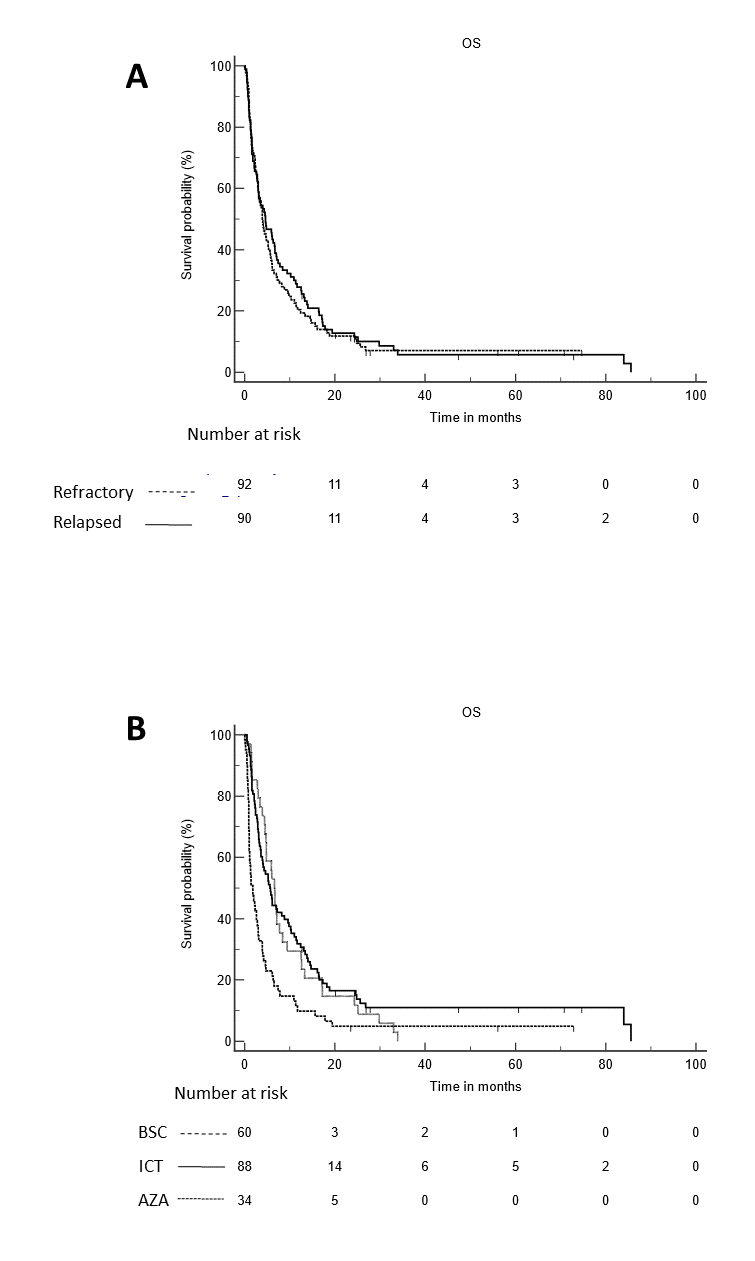


Figure S4.


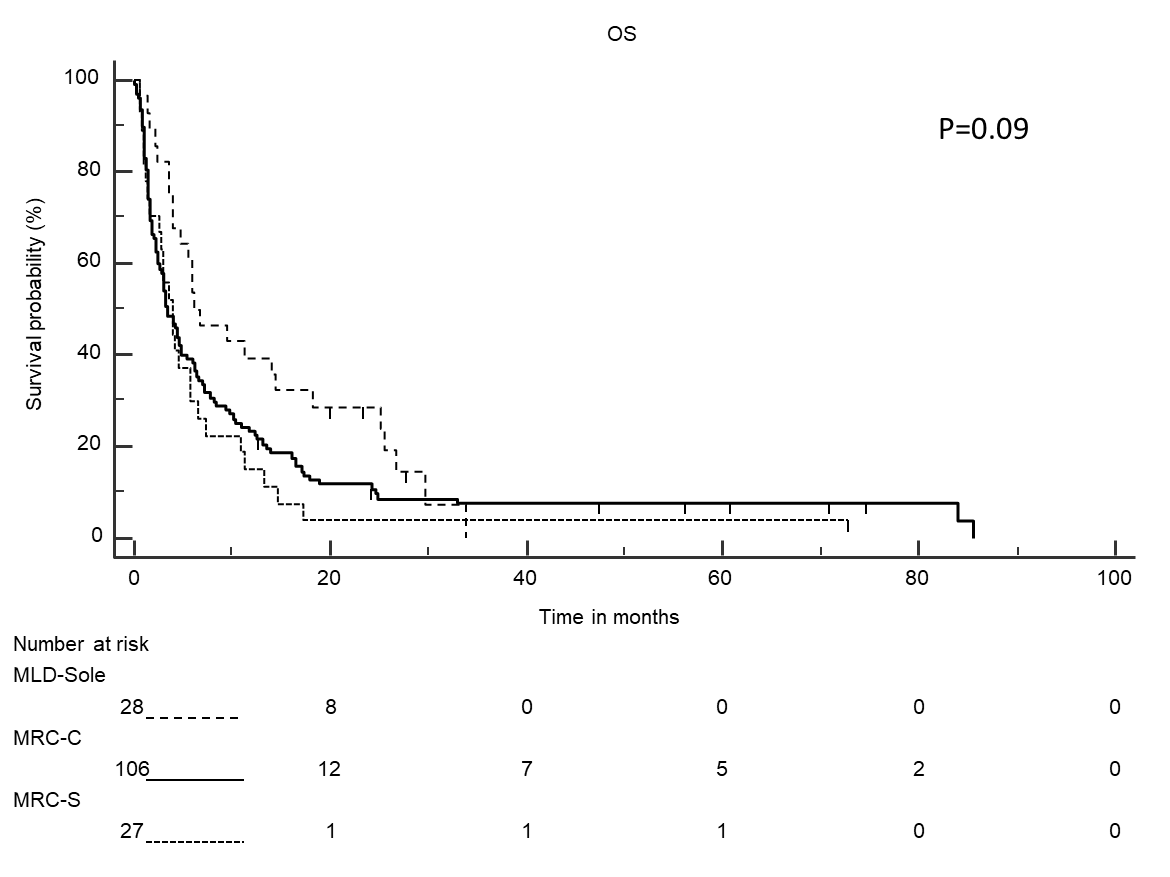


Figure S5.


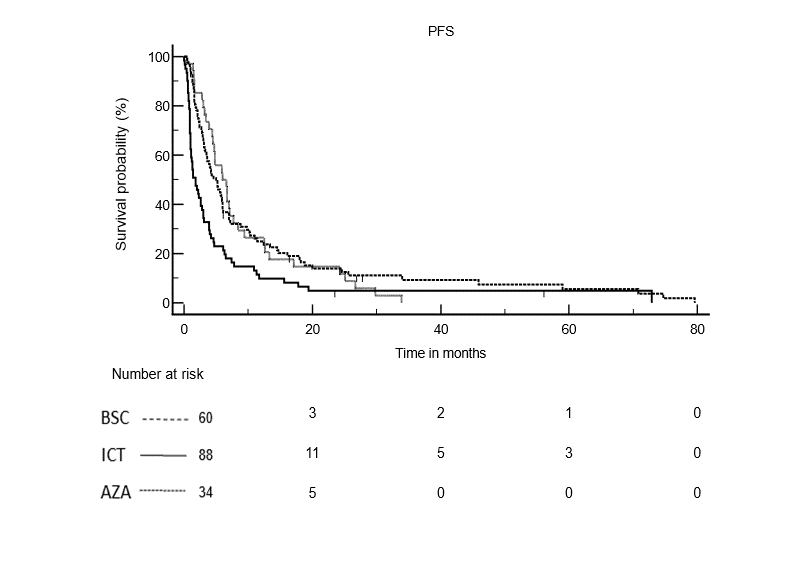

Supplement: Supplementary file 1 — Appendix S1. [file CAM4-13-e7003-s001.doc]
